# Maximum limit to the number of myosin II motors participating in processive sliding of actin

**DOI:** 10.1038/srep32043

**Published:** 2016-08-24

**Authors:** Khushboo Rastogi, Mohammed Shabeel Puliyakodan, Vikas Pandey, Sunil Nath, Ravikrishnan Elangovan

**Affiliations:** 1 Department of Biochemical Engineering and Biotechnology, Indian Institute of Technology, New Delhi 110016, India; 2 Department of Chemical Engineering, Indian Institute of Technology, New Delhi 110016, India

## Abstract

In this work, we analysed processive sliding and breakage of actin filaments at various heavy meromyosin (HMM) densities and ATP concentrations in IVMA. We observed that with addition of ATP solution, the actin filaments fragmented stochastically; we then determined mean length and velocity of surviving actin filaments post breakage. Average filament length decreased with increase in HMM density at constant ATP, and increased with increase in ATP concentration at constant HMM density. Using density of HMM molecules and length of actin, we estimated the number of HMM molecules per actin filament (*N*) that participate in processive sliding of actin. *N* is solely a function of ATP concentration: 88 ± 24 and 54 ± 22 HMM molecules (mean ± S.D.) at 2 mM and 0.1 mM ATP respectively. Processive sliding of actin filament was observed only when *N* lay within a minimum lower limit (*N*_*min*_) and a maximum upper limit (*N*_*max*_) to the number of HMM molecules. When *N* < *N*_*min*_ the actin filament diffused away from the surface and processivity was lost and when *N* > *N*_*max*_ the filament underwent breakage eventually and could not sustain processive sliding. We postulate this maximum upper limit arises due to increased number of strongly bound myosin heads.

Myosin II is a low duty ensemble motor that carries out physiological functions such as muscle contraction, actin cortex remodelling, and cytokinesis ring constriction. A single myosin II uses the free energy from ATP hydrolysis to slide an actin filament by 5–10 nm or to generate 5–10 pN force. During the acto-myosin ATPase cycle,myosin II remains attached to actin filament only for a small fraction of the total cycle time^1^. Under physiological conditions, for shortening or for development of tension, a large number of myosin molecules are required to function together in an ensemble.

The performance of group of myosin II molecules can be evaluated quantitatively using two properties: tension generation (T) and unloaded shortening velocity (*V*_*max*_). Shortening velocity and tension generation by a group of motors are inversely related i.e, at zero load myosin slide the filament at maximum velocity and at maximum load the filament velocity is zero. This force-velocity relationship has been well studied in single muscle cell^2^,^3^. However, it has been difficult to quantify the number of molecules participating at a particular load as there many parallel players in the sarcomere.

The advent of *in vitro* motility assay (*IVMA*)^1^,^4^,^5^,^6^ and 3-bead assay^7^,^8^,^9^, has enabled a more detailed understanding of mechanical and kinetic properties of acto-myosin interaction at single molecule level. *In vitro* under unloaded conditions, a single myosin molecule is capable of executing a power stroke on actin filament as force generated by a single head is greater than the drag experienced by the actin filament. For a low duty molecular motor such as myosin II, it has been a longstanding question in the field to quantify the shortening velocity as a function of the number of available motors and ATP. Pioneering classical work by the Spudich and Warshaw groups^1^,^10^ has shown that a minimum number of heads (*N*_*min*_) must be available for interaction with an actin filament for processive sliding of actin so that at least one myosin keeps the actin filament anchored at all times. As the number of available heads for interaction increase beyond *N*_*min*_, there is no further increase in filament sliding velocity. This has been explained as detachment-limited kinetics where filament velocity is limited by the rate of release of attached heads^11^. Recent experiments^12^,^13^,^14^ suggest that detachment kinetics, attachment kinetics and working stroke can be a function of size of the ensemble. This difference in mechanical and kinetic properties has been attributed to intermolecular/intramolecular force that they experience in ensemble motility.

For an evaluation of the ensemble behaviour of myosin II molecules, we have taken advantage of actin filament breakage and have quantified the number of motors involved in processive sliding in the classical *IVMA* at various heavy meromyosin (HMM) densities and ATP concentrations. We confirmed the earlier observations^15^,^16^,^17^,^18^ that actin filaments break into smaller fragments with addition of ATP in a stochastic process. With known HMM surface density and length of the sliding actin filaments we have estimated the number of HMM molecules (*N*) required for processive actin sliding. We make the novel finding that *N* is independent of the HMM density and depends *solely* on the ATP concentration. At 2 mM ATP, 64 ≤ *N* ≤ 112 (i.e. 88 ± 24) molecules and at 0.1 mM ATP, 32 ≤ *N* ≤ 76 (i.e. 54 ± 22) molecules are required for continuous sliding of actin in *IVMA*. We find that only those filaments that are able to interact with *N*_*min*_ ≤ *N* ≤ *N*_*max*_ HMM molecules slide continuously in the assay. If the number of interacting molecules per actin is less than the minimum limit, *N*_*min*_, then the actin filament is unable to sustain processive sliding. When the number of available HMM molecules per actin filament is greater than the maximum limit, *N*_*max*_, then the filaments are not able to sustain processive sliding and undergo breakage into smaller fragments. Actin filament breakage is consistent with a model in which strong binding of ‘*N*_*a*_’ HMM molecules on actin leads to a build-up of compressive stress between force-bearing heads and resisting rigor heads that eventually cause actin filament breakage by buckling.

## Results

### Protein purification and HMM density estimation

**Figure 1:**
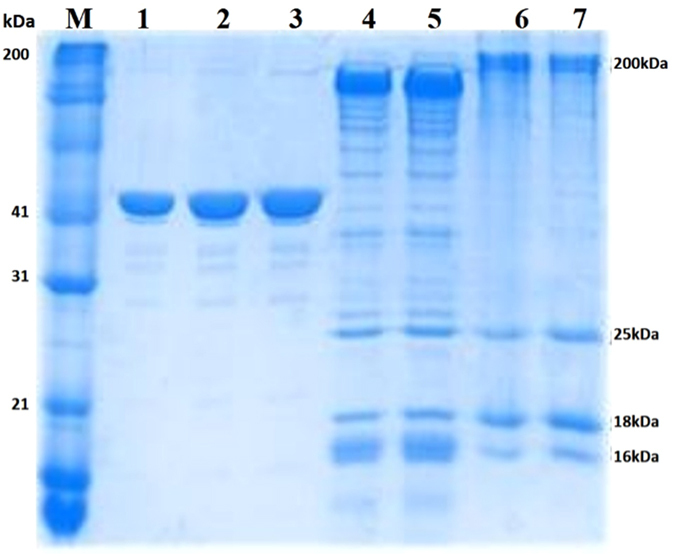
15% SDS-PAGE of myosin, HMM and actin from chicken pectoralis skeletal muscle. Lane 1,2,3 – G actin, Lane 4,5 –HMM, Lane 6,7 – Myosin (M-protein marker, Myosin heavy chain 200 kDa, HMM Heavy Chain – 170 kDa, LC –Light Chain 1 (26 kDa), Light Chain 2 (18 kDa), Light Chain 3 (16 kDa).

**Figure 2:**
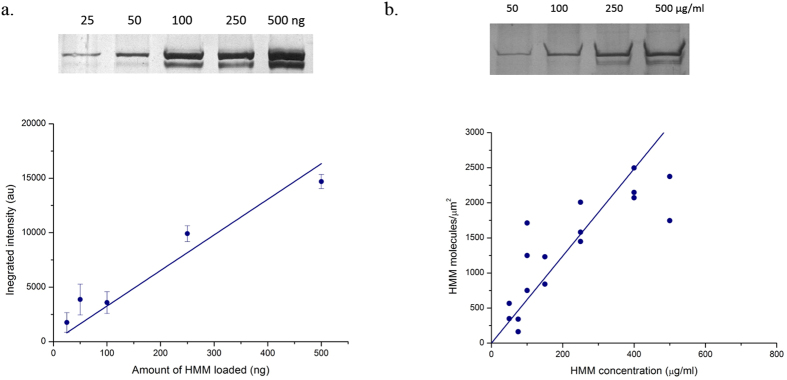
(**a**)Top: 8% SDS gel showing band with varying intensity depending on the amount loaded in the well (1000, 500, 250, 100, 50, 25 ng). Bottom: Relationship between the band intensity (au) and known amount of HMM. Bars indicate standard error. Linear fit is with slope 32.66 ± 3.68 and intercept set at zero. **(b)** Top: 8% SDS gel with eluate from incubated HMM concentration of 500, 250, 100, 50 μg/ml from surface density experiments. Bottom: Relationship between applied HMM concentration (μg/ml) and HMM molecules/μm^2^. The values for the number of HMM molecules/μm^2^ were calculated from the standard plot. Linear fit is for points between 50–400 μg/ml with slope 6.21 ± 0.47 and intercept set at zero.

As described in the Materials and Methods Section, we have purified myosin, actin and heavy meromyosin (HMM) from chicken pectoralis muscle. The purity and quality of the preparation was verified with 15% SDS-PAGE gel (Fig. 1). These isolated proteins did not exhibit proteolytic fragmentation and they performed well in IVMA assay with greater than 90% of the filaments sliding continuously for a period of more than 20 minutes. The number of HMM molecules immobilized on the nitrocellulose surface were quantified by silver staining after carefully extracting the molecules from the flow cell (Fig. 2 and Materials and Methods). The density of HMM molecules immobilized with 1 min incubation at various concentrations was linear up to 400 μg/ml HMM incubation solution and saturated thereafter (Fig. 2b).

### Effect of HMM density on actin filament breakage

**Figure 3:**
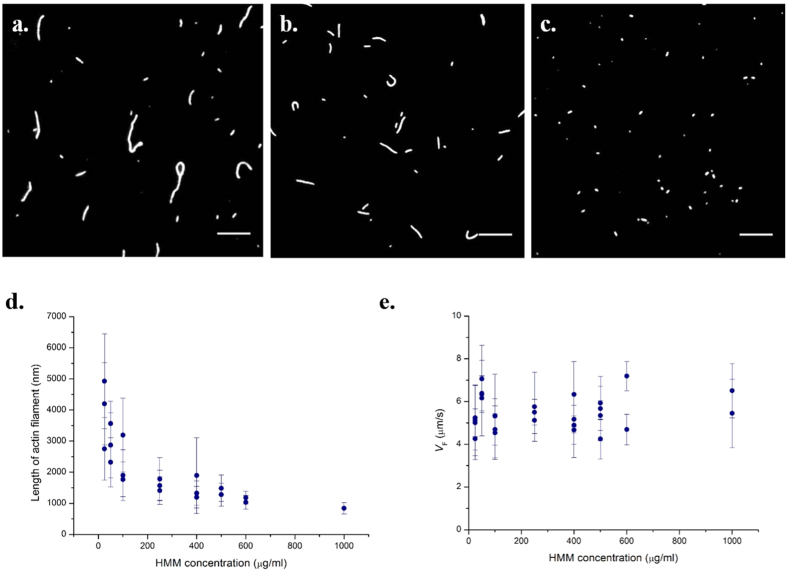
(**a**)Actin filaments before addition of ATP in *IVMA*. Most of the filaments are >5 μm. **(b,c)** Snapshot of *IVMA* at 25 μg/ml and 1000 μg/ml HMM density at 30 °C, 2 mM ATP. Scale bar corresponds to 5 μm. **(d)** Average length of the actin filaments sliding at different HMM density in an *IVMA*. **(e)** Actin sliding velocity (*V*_*F*_) as a function of HMM density. Actin sliding velocity was 5.5 ± 1.1 μm/s over the entire range of HMM density. Error bars denote standard deviation. All the data points in (**d**,**e**) correspond to a single IVMA experiment averaged from a minimum of 30 filaments satisfying our steady sliding criterion.

To study actin breaking and processive sliding, we performed all *IVMA* experiments with long actin filaments (average length 5–10 μm, Fig. 3a) before adding ATP at various HMM densities. With addition of ATP, the filaments started to slide and also broke into small fragments stochastically. In 5 minutes, most of the long filaments had broken and thereafter more than 70% of the filaments with their final steady state length exhibited continuous sliding. We found a very good correlation between the average length of filaments exhibiting steady state sliding and the HMM surface density. At high densities for example 600 μg/ml, the average length of sliding filaments was 1018.3 ± 201.3 nm, while at a low density such as 25 μg/ml, it was 3658.7 ± 1216.5 nm (Fig. 3b,c). As HMM surface density increased, the average length of sliding filaments was found to progressively decrease (Fig. 3d). The standard deviation of the average length of filaments was large at low HMM density while the variation in the length was minimum at saturating HMM density (>400 μg/ml). A histogram of the filament length distribution is shown in Figure S1.

Only actin filaments that moved steadily for a minimum distance of 2 μm were included in estimation of actin sliding velocity (*V*_F_). Between myosin densities of 25–1000 μg/ml, most of the actin filaments slid smoothly. We found that *V*_*F*_ was constant over the entire range of HMM density with an average *V*_*F*_ of 5.5 ± 1.1 μm/s at saturating ATP (2 mM) (Fig. 3e). However, at extremely low HMM density (<25 μg/ml), only a few filaments slid continuously. Most of the filaments showed a shorter run length, diffused from the surface, or exhibited random Brownian motion along the filament axis. These filaments were not included in the analysis since they did not represent unloaded myosin II shortening in the ensemble. However, we found that even the few filaments that slid continuously at these conditions had a velocity close to the average *V*_*F*_.

### Effect of [ATP] on actin filament breakage

**Figure 4:**
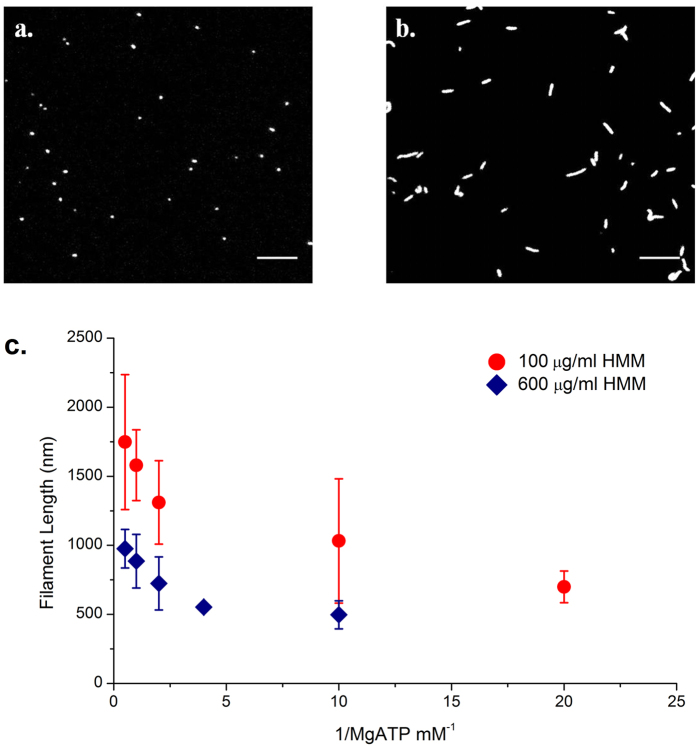
Snapshot of *IVMA* experiments at **(a)** 0.05 mM and **(b)** 2 mM ATP concentration at 100 μg/ml HMM density, 30 °C. At 2 mM ATP concentration, the average filaments length was 1747.6 ± 614.4 nm, and as ATP concentration was reduced to 0.05 mM the average length of filaments reduced to 698.8 ± 114.7 nm. Scale bar corresponds to 5 μm. **(c)** Length of actin filaments as a function of ATP at HMM density: 100 μg/ml (circles) and 600 μg/ml (diamonds). Bars indicate mean standard deviation from a minimum of three IVMA experiments each including at least 30 filaments.

To study the effect of ATP concentration on the breakage of actin, we performed IVMA at varying ATP concentrations at two different HMM densities, one saturating density (600 μg/ml) and the other at a sub-saturating condition of HMM density (100 μg/ml). For 100 μg/ml HMM density and 2 mM ATP concentration, the average length of sliding actin filament measured 1747.6 ± 488.6 nm. As the ATP concentration was decreased to 0.05 mM, the average length of actin filament reduced to 698.8 ± 114.7 nm. At high ATP concentration, we observed sliding of longer actin filaments while at low ATP concentration, only shorter filaments were found to exhibit continuous sliding (Fig. 4a,b). We obtained a similar decreasing trend in length of sliding filaments relation at both the HMM surface densities studied (Fig. 4c). At saturating HMM density and 2 mM ATP concentration, average filament length of processive actin measured 954.3 ± 126.2 nm, while the average filament length decreased to 599.5 ± 198.5 nm at 0.1 mM ATP concentration. At saturating HMM densities for ATP concentrations lower than 0.1 mM, we could not accurately determine the filament length (length < 0.5 micron) owing to the diffraction limit in resolution and the method adopted for length determination in this study. Hence they were omitted from the analysis.

To confirm that actin breakage was an ATP-dependent process, we exchanged the ATP solution in the flow chamber as a step jump. For example, after reaching steady filament sliding at 2 mM ATP, we exchanged the flow chamber solution with 0.1 mM ATP. Upon this exchange, the filaments exhibited further breakage and reached a smaller steady state filament length that showed smooth sliding. Following another exchange of flow chamber solution to the original concentration of 2 mM ATP, we found most of the filaments diffused away from the surface and were unable to sustain continuous motility. Rate of actin breakage was also dependent on the ATP concentration; at low ATP concentrations, filaments rapidly broke into smaller pieces. For example at 0.05 mM ATP more than 90% of the filaments fragmented within 60 s. This ATP-dependent sliding and breakage clearly demonstrates that actin breakage is due to the kinetics of the acto-myosin ATPase cycle that determines the number of motors participating in ensemble sliding. As a control we have also done the same experiments with full length myosin preparation. Actin breakage was also seen with full length myosin molecules and length distribution of actin filaments at different densities was very similar to HMM data (Figure S2).

### Number of HMM molecules participating in continuous actin sliding

**Figure 5:**
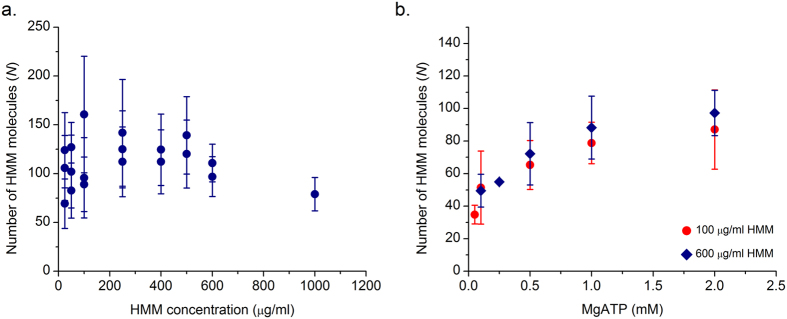
(**a**)Number of myosin molecules available for interaction: 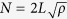, where *ρ* is the motor surface density and *L* is the actin filament length. Number of interacting myosin heads (*N*) available to bind to actin filaments at different HMM densities at saturating 2 mM [ATP]. Each point is average of 30 filaments from a single IVMA experiment. **(b)** Number of interacting myosin heads (*N*) available to bind to actin filament at different [ATP]. Values were determined at two different HMM densities: 100 and 600 μg/ml. Each point is an average of minimum 3 IVMA experiments with 30 filaments each.

Since the actin filaments were incubated in the presence of ATP for steady state equilibration, they experience a selection process because of breakage as described above. After the 5 min incubation no further change in the length of processively sliding filaments was found in all the experimental conditions studied. We determined the number of interacting heads from the measured steady state length of actin (Figs 3d and 4c) and the HMM surface density. We calculated the density of HMM molecules with SDS gel electrophoresis (Fig. 2b). Assuming a Poisson distribution of HMM molecules on the surface, the number of HMM molecules available for interaction with an actin filament can be calculated using the equation 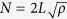, where *ρ* is the HMM surface density and *L* is the length of actin filament^10^. The length of the actin filament decayed monotonically with increase in HMM density (Fig. 3d). However, we observed that the number of HMM molecules that interact with an actin filament were almost constant over an HMM density of 25–1000 μg/ml. On the average, 90 HMM molecules were participating to continuously slide actin filaments at a saturating ATP concentration of 2 mM (Fig. 5a). The number of HMM molecules interacting with an actin filament can also be calculated by a band model^10^ using the equation *N* = *ρwL* where w, the width of the band is usually taken as 30 nm. We find that use of the band model leads to a lower estimate of the average number of HMM molecules interacting with actin filament compared to the nearest neighbour model at all conditions. Since we are primarily interested in defining the maximum limit, *N*_*max*_, the nearest neighbour model has been used throughout.

Similarly, we determined the required number of molecules for continuous sliding as a function of ATP concentration. The number determined at two different HMM densities overlapped each other within the range of experimental error. The number of HMM molecules participating in continuous sliding increased with increase in ATP concentration. The number of HMM molecules participating in continuous motility of actin at 0.1 mM ATP and 2 mM ATP was 54 ± 22 and 88 ± 24 (mean ± S.D.) molecules respectively (Fig. 5b). These numbers mean that we did not observe processive sliding of actin filament when the number of interacting HMM molecules were outside the ± range of the numbers cited above. These estimates allow us to arrive at a minimum lower limit (*N*_*min*_) and a maximum upper limit (*N*_*max*_) to the number of HMM molecules that can interact with a smooth sliding actin filament in IVMA under our experimental conditions. In summary, processive sliding of actin at *V*_*F*_ velocity requires the number of interacting HMM molecules to be in the range of *N*_*min*_ ≤ *N* ≤ *N*_*max*_.

### Sequence of Actin fragmentation

**Figure 6:**
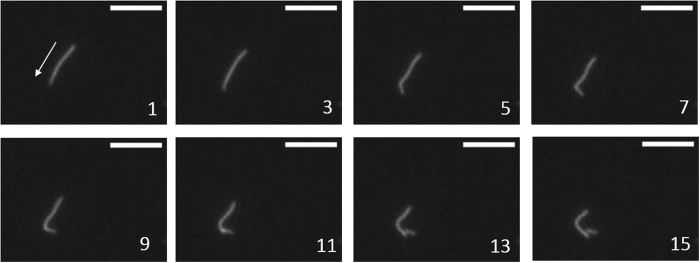
Example of actin breakage event imaged at 100 frames per second rate. Moving tip of the actin filament slows down and trailing end continues to move forward. Number indicates the order of image sequence, arrow in frame 1 shows the direction of sliding; scale bars correspond to 4 μm.

Actin fragmentation is a stochastic process, with the breakage kinetics dependent on the number of available heads over and above the maximum limit, *N*_*max*_. We found an incubation time of 5 minutes to be more than sufficient to reach the steady state sliding filament length and no further breakage was observed. The mechanism of actin breakage could be due to any of the following three different types of strains: (i) tug of war against a filament bound with dead myosin head, (ii) torsional load between two groups of molecules, (iii) buckling of actin filament between two groups of molecules. Study of several high-speed videos of actin filament breakage during the first few minutes revealed that most of them follow the sequence shown in Fig. 6. The leading tip of the sliding filament slowed down while the trailing end continued to move. We observed that this usually occurred when there is a bend along the axis of the filament. Next, a kink was formed along the filament that subsequently led to breakage of the actin filament i.e. by buckling mechanism (Fig. 6). The point at which the filament buckled was identified in the movies as a bright spot along the actin filament (Fig. 6, frame 11). S.K. Vogel *et al*.^18^ reported similar observations with myosin thick filament preparation at 0.3–1 μM ATP concentration.

### Actin sliding velocity dependence on [ATP]

**Figure 7:**
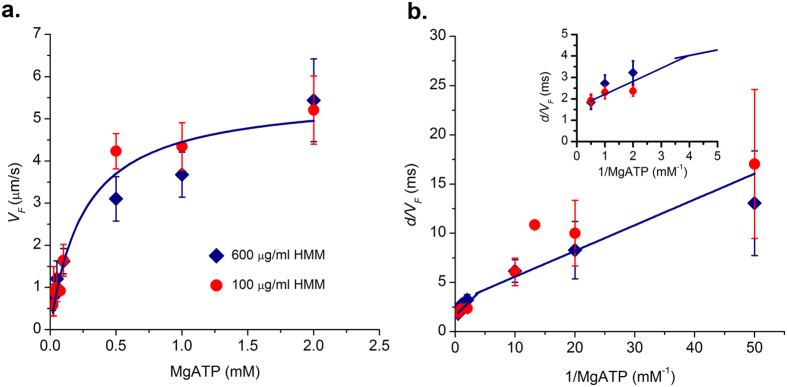
(**a**) Velocity of actin filament as a function of ATP at two different HMM densities; 100 μg/ml (circle) and 600 μg/ml (diamond). Solid line is Michaelis-Menten fit to filament velocity for a range of [MgATP]. *V*_*max*_ and *K*_*m*_ correspond to 5.5 ± 0.3 μm/s and 0.3 ± 0.03 mM respectively. **(b)** Double reciprocal plot of (**a**); value of d = 10 nm was used in the calculations. Data could be described using two linear fits for high and low ATP conditions. At high ATP (>0.5 mM) we calculated *k*_*−ADP*_ = 625 ± 139 s^−1^ and *k*_*+ATP*_ = 1.6 ± 0.14 × 10^6^ M^−1^ s^−1^ using equation 4. At low ATP (<0.5 mM) we calculated *k*_*−ADP*_ = 356.6 ± 74.6 s^−1^ and *k*_*+ATP*_ = 3.09 ± 0.24 × 10^6^ M^−1^ s^−1^. Inset shows the points and fit at high ATP for better visualization. Each point corresponds to an average from three different experiments with 30 filaments each. Error bars denote standard error.

We measured actin sliding velocities (*V*_*F*_) over a range of ATP concentrations (0.02–2 mM) at 100 μg/ml and 600 μg/ml (saturating) HMM concentrations. Each data point is the mean of four independent experiments, with each experiment containing a minimum of twenty trajectories. The actin sliding velocity increased with an increase in ATP concentration and reached a plateau at a saturated ATP concentration that follows Michaelis-Menten kinetics (Equation 1). Our data is plotted in Michaelis-Menten and double reciprocal Lineweaver-Burke representations (Equation 2)^14^,^19^ at the two different (100 μg/ml and 600 μg/ml) [HMM] respectively in Fig. 7a,b. The parameter values *V*_*max*_ and *K*_*m*_ determined at different densities overlapped and correspond to 5.5 ± 0.3 μm/s and 0.3 ± 0.03 mM respectively at (100 μg/ml and 600 μg/ml) HMM densities.


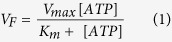



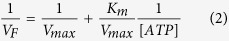



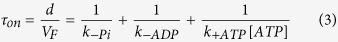



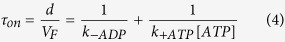


According to the detachment-limited model of actin sliding velocity, *V*_*F*_ = *d/τ*_*on*,_ where *d* is the myosin step size (assumed *d*~10 nm) and *τ*_*on*_ is the time myosin remains strongly bound to the actin filament. *τ*_*on*_ from ATPase cycle is given by equation 3, and is equal to the sum of the time taken for the three different kinetic steps i.e., 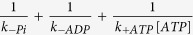, where 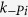 is the phosphate release rate, *k*_*−ADP*_ is the ADP release rate and *k*_+*ATP*_ is the second order rate constant for ATP binding. *k*_−*Pi*_ is a rapid step in the ATPase cycle (>1000 s^−1^)^20^ and has been ignored in equation 3 as the reciprocal value is negligible which leads to equation 4. Data in Fig. 7b could not be fitted using a single straight line, but could be described adequately using a biphasic plot. At high ATP (>0.5 mM), intercept yielded a value of *k*_−*ADP*_ = 625 ± 139 s^−1^ while the slope led to an estimate of *k*_+*ATP*_ = 1.6 ± 0.14 × 10^6^ M^−1^ s^−1^. Similarly, at low ATP (<0.5 mM) we calculated *k*_−*ADP*_ = 356.6 ± 74.6 s^−1^ and *k*_+*ATP*_ = 3.09 ± 0.24 × 10^6^ M^−1^ s^−1^. A similar biphasic slope has been obtained in the past for chicken and frog myosin preparations^15^,^19^. We did not find any difference in kinetic rate constants for two different HMM densities used in this study.

## Discussion

Actin breakage due to acto-myosin interactions has been reported by various groups^17^,^18^,^21^. Although breakage can occur due to inherent defects of the *IVMA*, e.g. presence of dead heads or random orientation of motors, here we show that breakage of actin filament can be induced by only changing the ATP concentration in a step exchange of flow chamber solution. This ATP-dependent actin fragmentation indicates that actin breakage is a direct consequence of the acto-myosin kinetics. In this study we have correlated the actin breakage to the number of myosin heads available for interaction.

All our *IVMA* experiments have been carried out with very long actin filaments before the addition of ATP. We found that upon incubation with ATP for a sufficient duration (5 minutes), all the long filaments break into smaller filaments, attaining a steady state length depending on HMM and ATP concentration. With experiments at two different densities and various ATP concentrations, we found the number of motors available is independent of the HMM density and is *solely* a function of ATP concentration. Though the steady state length of actin filaments is due to selection based on processivity, any filament that is above the steady state length eventually breaks down to smaller fragments. This indicates that the number of available motors dictates the actin breakage condition. As the number of motors available per actin filament (*N*) increases beyond the maximum upper limit *N*_*max*_, we found that the filament broke into smaller fragments. This maximum upper limit was found to be a function of ATP only.

It is unlikely that such a situation occurs in muscle, as there the myosin binding occurs in a 3D oriented lattice and further other stabilizing proteins anchor the actin filament in the half sarcomere. Also, *in vivo*, only 50 myosin molecules are oriented to interact with a single actin filament^22^; this value is smaller than the value of *N*_*max*_ observed in IVMA at saturating ATP. Moreover, our experiments with native thin filaments decorated with troponin-tropomyosin proteins do not exhibit breakage (manuscript in preparation), probably owing to the higher stiffness and more robust mechanical properties of the filament.

In our experiments, we found that breakage of actin occurs by a buckling mechanism i.e. kinking or bending of actin filament with a very small radii at a point along the axis of the filament. This was confirmed by observation of an increased fluorescence intensity spot at the point of actin breakage (Fig. 6). S.K. Vogel *et al*.^18^ also observed buckling-based actin breakage by the myosin thick filament at 0.3–1 μM ATP concentration; however actin breakage was not observed in those preparations above 10 μM ATP concentration. As the number of available motors are far lower in their thick filament preparation in comparison to the *IVMA* geometry, it might explain why breakage was observed only at low ATP concentrations.

Processive sliding of actin filament requires that at least one myosin head remains attached to the actin filament. To study the requirements of processive sliding, we took advantage of stochastic breakage of actin filament that were incubated long enough to maximize the number of breakage events. This breakage and selection process allows quantification of the number of heads available (*N*) for interaction with actin filament at various HMM densities and ATP concentrations (Fig. 5). Post breakage, only the filaments that are able to interact with a number of heads *N* within the range *N*_*min*_ ≤ *N* ≤ *N*_*max*_ heads continue to slide processively. For various HMM densities at saturating ATP, we estimate that 88 ± 24 HMM molecules are required to sustain processive sliding of actin filaments. This value correlates well with results from three other sources. a. Walcott, Warshaw *et al*.^13^ estimate ~200 heads are sliding the filament at maximum sliding velocity; b. Uyeda, Kron *et al*.^10^, determined the minimum length of filament required to slide filament at maximum velocity. From their length and density estimates we calculate that ~90 HMM molecules are required to sustain processive sliding at saturating ATP; c. Persson *et al*.^23^, determined 161 heads as the minimum number of myosin molecules required for maximum sliding velocity.

By varying ATP and density (Fig. 5b), we found that *N* is only a function of ATP and is independent of the HMM density. We estimated that at high ATP concentration (2 mM), the number of HMM molecules required for sustained sliding are 88 ± 24. At low ATP (0.1 mM), 54 ± 22 HMM molecules are sufficient to drive the steady state translocation of actin. As the time of attachment (*τ*_*on*_) increases with decrease in the ATP concentration, there is more time for each myosin head to search a binding site on actin stochastically, become strongly bound and then proceed with an almost instantaneous working stroke. Thus, fewer molecules are capable of sliding actin continuously at low ATP concentrations (e.g., 0.1 mM) compared to saturating ATP, all other conditions remaining the same. A similar observation was also made by Sato *et al*.^24^ in their experiments that the processivity of actin filament depends on ATP concentration and HMM density and actin filaments slide for longer distance at low ATP and high HMM density.

**Figure 8:**
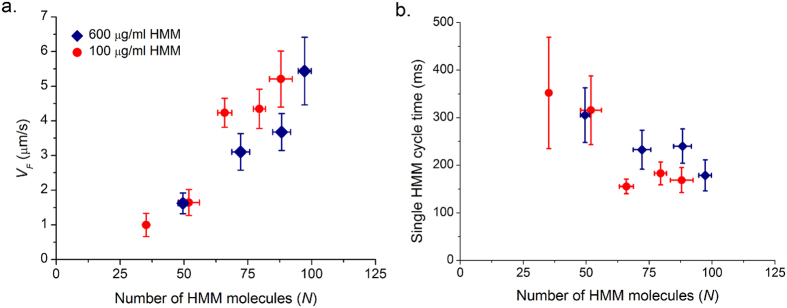
(**a**) Velocity of actin filaments (*V*_*F*_) as function of ATP from Fig. 7a and number of HMM molecules (*N*) available for interaction as function of ATP from Fig. 5b are plotted together at two different HMM densities (100 and 600 μg/ml). **(b)** Assuming a working stroke of *d* = 10 nm, with known number of interacting motors (*N*) and average sliding velocity (*V*_*F*_) we calculated number of working strokes per molecule per second or average time taken for single ATPase cycle by a HMM molecule (*N*. *d/V*_*F*_). Average cycle time is plotted as function of number of motors available for interaction. Error bars denote standard error.

We have plotted *V*_*F*_ as a function of ATP from Fig. 7a and *N* as a function of ATP from Fig. 5b together in Fig. 8 i.e., measured *V*_*F*_ and *N* at a given ATP. *V*_*F*_ observed in *in vitro* motility assay is limited by available ATP concentration. The number of motors available for interaction is a function of ATP as well. There is a strong linear correlation between *V*_*F*_ and *N* interacting per actin filament at a given ATP (Fig. 8a). In our assay, the actin filaments slide at a steady velocity of 5.5 μm/s (Fig. 3e) at 2 mM ATP. Assuming a working stroke of 10 nm per acto-myosin interaction, at least 550 myosin heads must interact every second. Given there are *N* molecules available per actin, we calculated the average time taken for a single ATP cycle per myosin molecule *N.d/V*_*F*_ (Fig. 8b). At 2 mM ATP, average time taken for a single ATP cycle is ~135 ms and average time a single myosin remains attached to actin filament *τ*_*on*_ ~ 1.8 ms from Fig. 5b. After detachment, the myosin motor hydrolyses the ATP molecule in ~10 ms and re-primes to pre-power stroke conformation^25^. If we assume instantaneous binding after hydrolysis step, each molecule is capable of making 85 working strokes per second, only 7 HMM molecules are required for executing 550 working strokes per second. However we estimate a minimum of *N*_*min*_ = 88–24, i.e. 64 molecules for continuous steady state actin motility at saturating ATP, much larger than the above idealized case. In *IVMA,* the HMM molecules are randomly oriented and the immobilization of HMM might hamper the free movement of the heads in comparison to *in vivo* systems.

To understand further details of the actin breakage mechanism, it is important to determine the number of motors simultaneously attached during unloaded shortening. So far, there exists no experimental evidence for estimation of the number of motors attached simultaneously in *in vitro* assays. We can however infer the number of attached heads using published duty ratio values; for example a duty ratio of about 0.02 has been reported for ensemble motility^1^,^13^. Using this duty ratio value, we can calculate the number of strongly bound molecules *N*_*a*_ to be ~1 at *N*_*min*_ and ~2 at *N*_*max*_ at saturating ATP concentrations. There are recent reports^12^,^23^ suggesting that *N*_*a*_ could be as high as 6–8 during unloaded shortening. The number of simultaneously attached heads during unloaded shortening raises many other questions such as how *τ*_*on*_ depends only on ATP concentration. These questions require a deeper understanding of the mechano-chemical aspects of the catalytic cycle of double-headed myosin, and especially of the key role of myosin activation and the physical constraints associated with the force-generation step in muscle contraction^13^,^26^,^27^.

In an ensemble, it is intuitive to think that as the number of interacting motors increase, the output of the system would be additive. However this is not the case, for example *in vivo* there is a maximum limit to unloaded shortening velocity even with increasing overlap between thick and thin filament^3^ and there is maximum to isometric tension developed with fixed number actin binding sites as a function of pCa^28^. These maximum performance limitations are due to intrinsic rate limiting steps in the acto-myosin ATPase cycle and the mechanical properties of actin and myosin molecules. To the best of our knowledge, the finding of an upper maximum limit to the number of participating motors has not been reported earlier at zero load conditions as in *IVMA*. We believe that this new concept could also be applicable to other low-duty ratio motors. It would be interesting to evaluate if other non-processive motors with different duty ratios exhibit similar limitations.

## Materials and Methods

### Protein purification

Fast skeletal heavy meromyosin was prepared from chicken pectoralis muscle obtained from local poultry shop. The tissue was cut into small pieces and stored in ice chilled incubation buffer (Potassium phosphate Buffer 170 mM, EGTA 5 mM, Na_2_ATP 2.5 mM, MgCl_2_ 5 mM, PMSF 10 μM at pH 7.0). The tissue was minced and homogenized in extraction buffer (Potassium phosphate Buffer 150 mM, KCl 300 mM, MgCl_2_ 5 mM, Na_2_ATP 2.5 mM, Na_4_P_2_O_7_ 10 mM, DTT 10 mM, PMSF 10 μM at pH 6.6) and incubated for 15 minutes with constant stirring. Myosin dissolved in the extraction buffer was purified using multiple steps of polymerization-depolymerization method developed by Margossian and Lowey (1982)^29^. In last step, the myosin filaments, collected via centrifugation at 10,000 g for 20 minutes, were resuspended in a depolymerizing buffer (KCl 600 mM, MOPS 20 mM, MgCl_2_ 5 mM, Na_4_P_2_O_7_ 2 mM, βME 1% at pH 7.2). HMM was prepared from this freshly prepared myosin using α-chymotrypsin digestion as described by Guo *et al*.^30^. All steps were performed at 4 °C. Isolated HMM was stored at −20 in 50% glycerol v/v and used for two weeks.

G-actin was purified from chicken skeletal muscle following the protocol of Pardee and Spudich, (1982)^31^ and stored at −80 °C and used for one year. G-actin was polymerized to F-actin and fluorescently labelled with five molar excess of Tetra methyl rhodamineisothiocyanate phalloidin (TRITC)according to the method of Kron *et al*.^6^.

Protein concentration for actin, myosin, and HMM was determined using Bradford assay (Bradford, 1976)^32^. The purity of the final protein preparation was confirmed with 15% sodium dodecyl sulphate-polyacrylamide gel electrophoresis (SDS-PAGE) and stained by Coomassie brilliant blue R-250.

### *In vitro* motility assay

The *IVMA* was performed as described by Kron and Spudich *et al*.^6^,^19^. Flow through chambers were made using 0.1% nitrocellulose coated cover slips and glass slides using double sided sticky tape. Assay buffer contained KCl 25 mM, Imidazole 25 mM, EGTA 1 mM, MgCl_2_ 4 mM, DTT 10 mM, pH 7.5 at 30 °C. HMM solution with concentration ranging from 25–1000 μg/ml was added to flow chamber and incubated for 60 seconds. Precisely, after 60 seconds, unbound HMM molecules were washed by assay buffer with BSA (0.5 mg/ml). BSA wasused to block free nitrocellulose surface to minimize nonspecific binding. After 3 minutes incubation, 60 μl of TRITC labelled actin (7 nM) was added to the chamber and incubated for 60 seconds. Care was taken to avoid shearing of actin at this step. After actin incubation, a reaction mixture with 2–0.02 mM ATP, 0.5% methyl cellulose, an oxygen scavenger system (0.2 mg/ml glucose oxidase, 0.05 mg/ml catalase, 3 mg/ml glucose) in assay buffer was exchanged in flow chamber. For low ATP experiments, an ATP regeneration system was added (creatine phosphate 10 mM, creatine phosphokinase 0.1 mg/ml). Depending on ATP concentration in reaction mixture, KCl was varied between 18–38 mM to maintain the ionic strength at 60 mM and free Mg^2+^ concentration was always maintained at 1 mM. Amount of KCl and MgCl_2_ required were obtained using a custom program similar to those described elsewhere^33^,^34^. After addition of reaction mixture, the flow chamber was immediately mounted on a temperature controlled trough maintained at 30 °C. The slide was incubated for five minutes before data acquisition.

### Microscopy and image analysis

Sliding of actin filaments was imaged with an inverted fluorescence microscope (Olympus IX 71) with 100 W mercury lamp and Andor Neo sCMOS camera. The fluorescent signal collection was maximized by using a large NA objective (60X, 1.4 NA), filters and dichroic mirrors with high quantum yield and transmission spectra optimized for TRITC. Movies were acquired with 50 ms exposure. For measuring velocities at low ATP concentrations, a delay time of 150 ms and 450 ms was introduced between the frames to prevent underestimation of filament velocity due to truncation of filament paths. Movies of sliding filaments were captured at different position and stored in computer for further analysis. Movies were analysed using MTack 2 plugin (http://valelab.ucsf.edu/~nstuurman/IJplugins/MTrack2.html) in image J http://rsb.info.nih.gov/ij/and tracks were identified using a custom built program in LabVIEW (National Instruments). Only filaments that were at least 0.5 μm, moved >2 μm and a consistent velocity (standard deviation/mean velocity <0.5) were included in data sets. Per frame velocity from 20–30 tracks was pooled, and mean and standard deviation were obtained by fitting a Gaussian curve. Actin filament length was determined by measuring the pixels between the two ends of the filament in *Image J*.

### Measurement of HMM head density on the coverslip

HMM density at various conditions were measured by analysing the amount of immobilized HMM molecules using SDS-PAGE followed by quantification using silver staining^24^. Flow chamber was made with two cover slides, both coated with 0.1% nitrocellulose. Varying incubation concentrations of HMM (0.025–0.5 mg/ml) were added to flow chamber, and after 60 seconds incubation, unbound HMM molecules were washed with assay buffer. The immobilized HMM in the coverslip was eluted with 100 μl sample buffer (1% SDS, 6.25 mM Tris-HCl pH 6.8, 10% glycerol, and 10% 2-β mercaptoethanol). The eluent was passed multiple times to recover all HMM bound to the surface. This eluate was loaded in 8% SDS gels and electrophoresis was performed. The gel was stained using ProteoSilver^TM^ silver stain kit (Sigma Lifesciences) and band intensities were analysed with *ImageJ*.

## Additional Information

**How to cite this article**: Rastogi, K. *et al*. Maximum limit to the number of myosin II motors participating in processive sliding of actin. *Sci. Rep.* **6**, 32043; doi: 10.1038/srep32043 (2016).

## Supplementary Material

Supplementary Information

Supplementary Video

Supplementary Video

Supplementary Video

Supplementary Video

Supplementary Video
