# Supplementary material for: Maximum limit to the number of myosin II motors participating in processive sliding of actin
Source: Sci Rep. 2016 Aug 24;6:32043. doi: 10.1038/srep32043 (PMC4995457; doi:10.1038/srep32043)
Supplement: Supplementary Information [file srep32043-s1.pdf]

Supplementary information

Maximum limit to the number of myosin II motors participating in processive sliding of actin

Khushboo Rastogi[1], Mohammed Shabeel Puliyakodan[1], Vikas Pandey[2], Sunil Nath[1] and Ravikrishnan Elangovan[1,*]

[1] Department of Biochemical Engineering and Biotechnology,

Indian Institute of Technology, New Delhi, India.

[2] Department of Chemical Engineering,

Indian Institute of Technology, New Delhi, India.

Distribution of actin filament length at various conditions

Actin filament length data shown in Figure 3 and Figure 4 are pooled and plotted as histograms for different ATP concentrations and HMM densities (Figure S1).

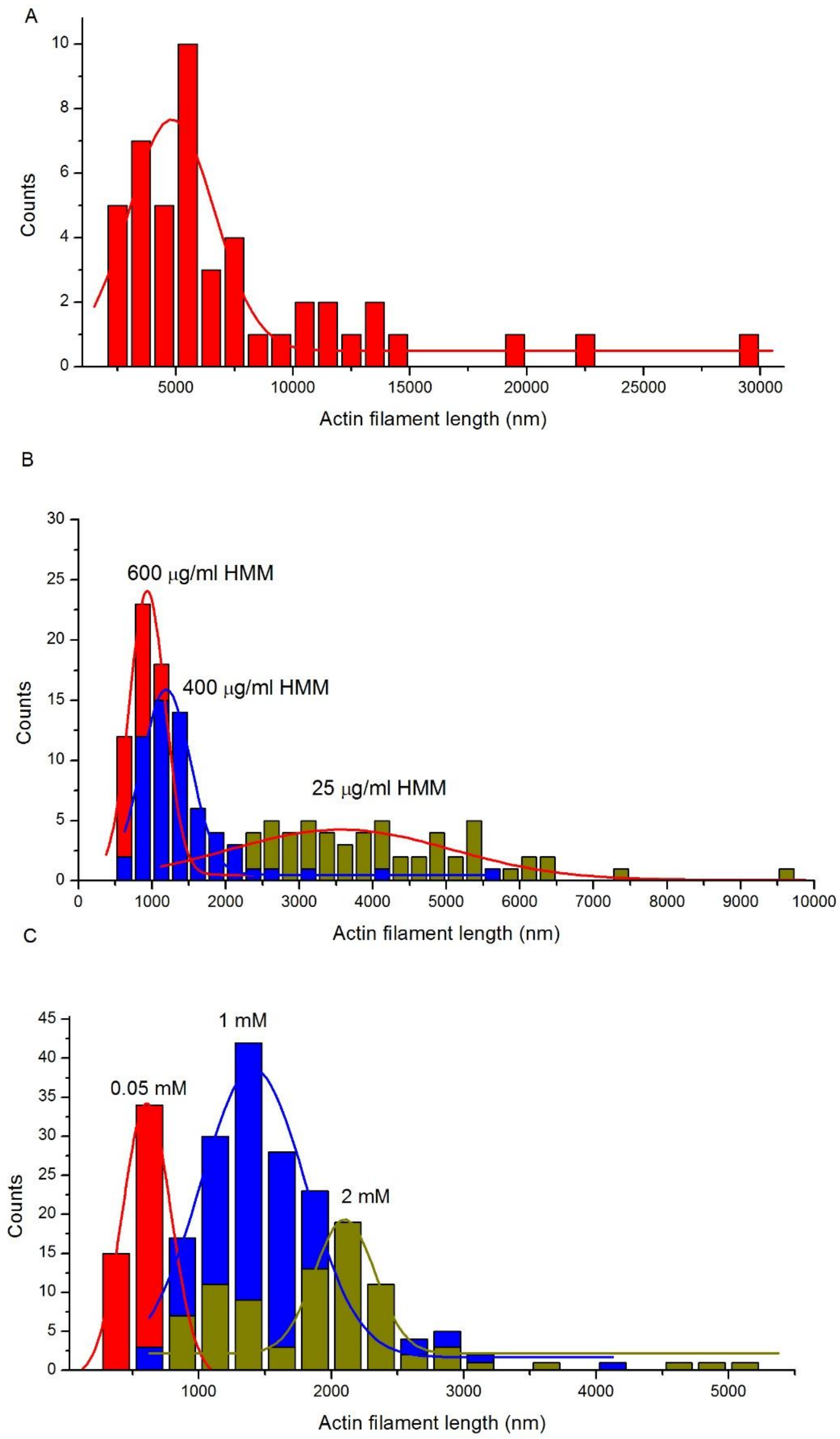
A
Counts
Actin filament length (nm)
B
600 μg/ml HMM
400 μg/ml HMM
25 μg/ml HMM
Counts
Actin filament length (nm)
C
0.05 mM
1 mM
2 mM
Counts
Actin filament length (nm)

Figure S1: A. Histogram of actin filament length before the addition of ATP. Solid line is Gaussian fit with 4.8±1.8 µm (µ ± σ) (Eq. 1). The bin size was 1000 nm. B. Histograms of actin length distribution for 2mM ATP and various HMM density conditions. Gaussian fit results for 600, 400, and 25 µg/ml HMM are 0.9 ± 0.24, 1.2 ± 0.3, and 3.6 ± 1.5 µm respectively. The bin size was 250 nm. C. Histograms of actin length distribution for 100 µg/ml HMM density and various ATP concentrations. Gaussian fits for 0.05 mM, 1 mM, and 2 mM ATP are 0.6 ± 0.2, 1.4 ± 0.4, and 2.1 ± 0.2 µm respectively. The bin size was 250 nm. A minimum 60 data points were used for analysis of each histogram.

Actin breakage using full length myosin preparation

We have repeated the experiments with full length myosin preparation using protocol similar to that described in Elangovan et al[1]. IVMA experiments were carried out exactly as for HMM preparations, with an additional step for blocking dead heads. Dead heads were blocked by adding unlabeled fragmented actin (prepared by sonication for 30 s) and subsequent washing with 1 mM ATP solution. Results were very similar to HMM preparations (Figure S2).

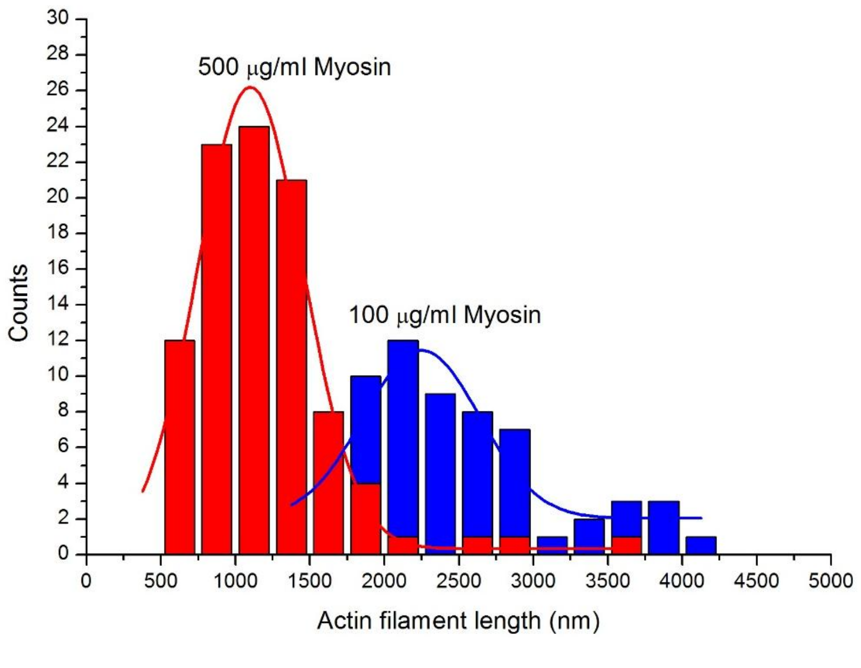

Figure S2. Histograms of actin length distribution for myosin molecules at 2 mM ATP and various motor densities. Solid line is Gaussian fit (Eq. 1). Results for 500 µg/ml and 100 µg/ml myosin densities are 1.1 ± 0.35 and 2.0 ± 0.4 µm respectively. The bin size was 250 nm.

Movie S1. Movie of IVMA with 1000µg/ml HMM concentration and 2mM ATP. Movie acquired at 20 fps.

Movie S2. Movie of IVMA with at low HMM density (25µg/ml) and 2mM ATP. Movie acquired at 20 fps.

Movie S3. Movie of IVMA with 100µg/ml HMM density at 2mM ATP concentration. Movie acquired at 20 fps.

Movie S4. Movie of IVMA with 100µg/ml HMM density and 0.025mM ATP. Movie acquired at 20 fps.

Movie S5. Actin filament breakage at 250µg/ml HMM concentration at 2mM ATP concentration. Movie acquired at 100 fps and displayed at 20 fps.

Reference

1 Elangovan, R. *et al.* An integrated in vitro and in situ study of kinetics of myosin II from frog skeletal muscle. *J Physiol* **590**, 1227-1242, doi:10.1113/jphysiol.2011.222984 (2012).
